# Heterologous Fibrin Biopolymer as a Key Scaffold for Bone Regeneration: Synergistic Effects with Photobiomodulation and Membrane Therapy

**DOI:** 10.3390/gels12010056

**Published:** 2026-01-06

**Authors:** Matheus Bento Medeiros Moscatel, Bruna Trazzi Pagani, Beatriz Flávia de Moraes Trazzi, Tawana Pascon, Benedito Barraviera, Rui Seabra Ferreira Júnior, Daniela Vieira Buchaim, Rachel Gomes Eleutério, Rogerio Leone Buchaim

**Affiliations:** 1 Postgraduate Program in Applied Dental Sciences, Bauru School of Dentistry (FOB/USP), University of Sao Paulo, Bauru 17012-901, Brazil; matheusbmm.96@usp.br (M.B.M.M.); brunatrazzi@usp.br (B.T.P.); 2 Dentistry School, University of Marilia (UNIMAR), Marilia 17525-902, Brazil; flavia.odonto@unimar.br (B.F.d.M.T.); tawanapascon@unimar.br (T.P.); rachel.ge@unimar.br (R.G.E.); 3 Center for the Study of Venoms and Venomous Animals (CEVAP), São Paulo State University (Univ. Estadual Paulista, UNESP), Botucatu 18610-307, Brazil; benedito.barraviera@unesp.br (B.B.); rui.seabra@unesp.br (R.S.F.J.); 4 Medical School, São Paulo State University (Univ. Estadual Paulista, UNESP), Botucatu 18618-687, Brazil; 5 CTS-CEVAP (Center for Translational Science and Biopharmaceutical Development), São Paulo State University (Univ. Estadual Paulista, UNESP), Botucatu 18610-307, Brazil; 6 Graduate Program in Anatomy of Domestic and Wild Animals, Faculty of Veterinary Medicine and Animal Science, University of Sao Paulo, Sao Paulo 05508-270, Brazil; danibuchaim@alumni.usp.br; 7 Medical School, University Center of Adamantina (FAI), Adamantina 17800-000, Brazil; 8 Department of Postgraduate, School of Dentistry, Faculty of Midwest Paulista (FACOP), Piratininga 17499-010, Brazil; 9 Department of Biological Sciences, Bauru School of Dentistry (FOB/USP), University of Sao Paulo, Bauru 17012-901, Brazil

**Keywords:** bone repair, fibrin sealant, low-level laser therapy, photobiomodulation, regenerative medicine, guided bone regeneration, fibrin gel, scaffolds

## Abstract

Bone regeneration remains a clinical challenge, particularly in critical-size defects, motivating the investigation of biomaterials and adjuvant therapies that may support tissue repair. This experimental study evaluated bone healing in critical-size calvarial defects created in rats, using different combinations of regenerative strategies, including heterologous fibrin biopolymer gel, bovine cortical bone biological membrane, and photobiomodulation. Standardized 5.0 mm calvarial defects were surgically created in sixty Wistar rats, which were randomly allocated into six experimental groups according to the filling material and the application or absence of photobiomodulation. The treatments included clot alone, fibrin biopolymer gel, biological membrane, photobiomodulation, or their respective combinations. Animals were euthanized at 14 or 42 days, and bone repair was evaluated by histomorphometric analysis. At 14 days, differences in the extent of newly formed bone were observed among the experimental groups, with higher bone formation values detected in groups receiving combined treatments and lower values in groups treated with fewer regenerative components. At 42 days, all groups showed progression of bone repair, with greater bone formation observed in groups in which a biological membrane was used, regardless of photobiomodulation. Overall, the findings indicate that the association of different regenerative approaches was related to variations in bone repair patterns over time, suggesting that photobiomodulation, when applied in combination with biomaterials, may be associated with differences in early bone healing, without implying a direct causal effect.

## 1. Introduction

Bones play a fundamental role in the body and are responsible for supporting it and assisting in the performance of a wide range of functions and activities in everyday life [1,2,3,4]. It is worth noting that, depending on the type of activity or function required, bone tissue may be subjected to stimuli that promote fracture or resorption processes [5,6,7,8,9], which can lead to situations in which the volume and thickness of certain structures are altered or outside the normal range [10,11]. Under appropriate biological and mechanical conditions, natural bone repair is sufficient to completely restore the integrity of the skeleton in most fractures [12,13]. However, certain cases require correct intervention in order to enhance physiological repair mechanisms, with the aim of regenerating large amounts of bone [14,15,16].

The use of biomaterials in the medical and dental fields has been gaining increasing importance as the need to restore areas with bone defects or loss due to compromised morphology, function, and repair of biological tissues [17,18,19,20,21,22]. Bone grafts can be divided into autogenous, homogenous, xenogenous, and alloplastic. The most commonly used for block grafting are autogenous grafts, which are considered the “gold standard” due to their high incorporation potential, and homogenous grafts, given the impossibility of autogenous grafts, as well as their osteoconductive capacity [23,24,25]. Xenografts are of animal origin and have great osteoconductive potential [26,27,28,29]. These materials provide a scaffold that supports osteoblast migration and maintains space for the new bone formation, while presenting a low risk of contamination and physicochemical properties comparable to those of human bone, and are therefore commonly used in combination with other biomaterials to enhance regenerative outcomes [26,27,28]. Lastly, alloplastic grafts can also be listed, which are purely synthetic biomaterials produced in laboratories, being a product of standardized quality, with no risk of infectious diseases [30,31,32], composed largely of substances essential for the bone regeneration process, such as calcium or phosphorus [33,34].

Guided bone regeneration (GBR) has been incorporated as a therapeutic modality that aims to promote the neoformation of resorbed bone tissue through the application of membranes and biomaterials [35,36,37]. The concept of GBR was established based on the principle of guided tissue regeneration, whereby certain tissues regenerate when cells with this capacity populate the defect during the healing period [38]. Biological membranes from cattle have shown satisfactory results when used in experimental studies [39,40,41,42,43]. Their main functions are to provide structure for the orientation and development of new tissues through repair processes to restore the structure and function of the affected organ [40,44,45]. The GenDerm^®^ membrane (Baumer, Mogi Mirim, Brazil) is a bovine-derived cortical bone membrane characterized by a porous and acellular structure, high biocompatibility, and resorbable behavior, obtained through a controlled manufacturing process that ensures purity, absence of antigenic or pyrogenic components, and minimal contamination, while providing flexibility and ease of handling after hydration, with complete resorption occurring within approximately 45 days [46].

Various biomaterials have been developed for a wide range of purposes in the field of healthcare, one of which is the Heterologous Fibrin Biopolymer (CEVAP/UNESP, Sao Paulo State University, Botucatu, Brazil) [47]. The biopolymer is formulated from a thrombin-like serine protease isolated from *Crotalus durissus terrificus* venom combined with a fibrinogen-rich cryoprecipitate derived from *Bubalus bubalis* blood, which together enable fibrin network formation upon activation [48,49,50,51]. After mixing its components, the material undergoes rapid polymerization, forming a dense and translucent fibrin gel that mimics the final step of the coagulation cascade and results in a robust fibrin network [51,52]. This gel provides a versatile scaffold with hemostatic capacity, strong tissue adhesion, biodegradability, and potential for drug delivery [53,54], without requiring antifibrinolytic agents to maintain its stability [55]. The high fibrinogen content of the bubaline cryoprecipitate contributes to superior mechanical performance compared to human-derived fibrin sealants [56], supporting its increasing use in experimental models [57,58,59,60].

With regard to photobiomodulation, several studies demonstrate its potential for use in various fields such as medicine and dentistry, showing promising and favorable results [61,62,63,64,65,66,67,68]. According to the literature, electromagnetic radiation from lasers acts on photoreceptors present in the constitution of cells, promoting a photochemical interaction that has the potential to induce an increase in cellular metabolism, which in turn generates a series of biological effects such as analgesia, anti-inflammatory effects, and biostimulatory effects [62,63,69].

Despite the growing body of evidence supporting the individual use of biomaterials, barrier membranes, and photobiomodulation in bone regeneration, there is still limited understanding regarding the combined effects of these strategies when applied simultaneously in critical-size bone defects [70,71,72]. In particular, studies evaluating heterologous fibrin biopolymer as a three-dimensional scaffold associated with guided bone regeneration membranes and photobiomodulation remain scarce, especially in standardized preclinical models [73,74]. The interaction between a fibrin-based scaffold capable of promoting cell adhesion and matrix organization, the space-maintaining role of biological membranes, and the potential modulatory effects of photobiomodulation on the bone repair microenvironment has not been fully elucidated [75,76,77,78]. Therefore, investigating these combined approaches is relevant to clarify whether their association may influence bone repair patterns over time, contributing to the optimization of guided bone regeneration protocols and advancing translational perspectives in oral and maxillofacial bone reconstruction [79].

Given the above, it is clear that all techniques used to intervene in different manners play an important and complex role in the process of bone healing. In this context, the aim of this study was to evaluate, in a standardized rat calvarial critical-size defect model, the bone repair patterns associated with the use of heterologous fibrin biopolymer gel, bovine cortical bone biological membrane, and photobiomodulation, applied alone or in combination, through histomorphometric and collagen birefringence analyses at 14 and 42 days.

## 2. Results and Discussion

The experimental procedures were successfully performed according to the planned protocol, with all animals completing the designated postoperative periods. Throughout the study, no complications or adverse events were observed that could compromise the healing process or data collection. At 14 and 42 days, samples of the skull cap were obtained, processed as described in the Materials and Methods, and evaluated through histological, histomorphometric, and collagen birefringence analyses, allowing a detailed assessment of bone repair in the critical bone defects created. The results are presented below based on descriptive histological and quantitative analyses, followed by contextual interpretation in light of existing literature.

### 2.1. Histomorphometric Analysis

At 14 days, based on histological evaluation of the sections prepared and stained as described in Section 4, all groups showed cortical discontinuity with well-defined margins and partial filling of the defect by connective tissue. In the fibrin biopolymer group (FG), bone repair was limited, with the defect area largely occupied by loosely organized connective tissue and only occasional immature trabeculae observed at the peripheral margins of the defect. The photobiomodulation with fibrin biopolymer group (PFG) showed slight improvement compared to FG, showing initial trabeculae, but still poorly organized. The control group (CG) showed slightly more evident bone formation, although still limited to the margins of the defect. The group treated with biological membrane (CMG) showed initial bone deposition associated with more organized connective tissue, as observed in the histological sections, suggesting a stabilizing effect of the membrane on the regenerative microenvironment. In contrast, groups with biomaterial associations, such as fibrin biopolymer with biological membrane (FMG), showed greater deposition of immature bone adjacent to the margins. Based on the descriptive and quantitative findings presented above, the following interpretation considers the observed patterns in relation to previous experimental evidence.

**Figure 1:**
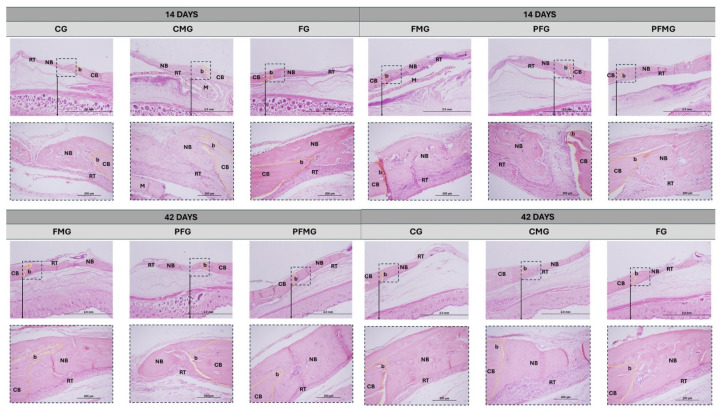
Hematoxylin–eosin–stained longitudinal sections of calvarial bone defects evaluated at 14 and 42 days. Control group (CG), fibrin biopolymer group (FG), clot and biological membrane group (CMG), fibrin biopolymer with biological membrane group (FMG), photobiomodulation with fibrin biopolymer group (PFG) and photobiomodulation, fibrin biopolymer, and biological membrane group (PFMG). Cortical bone (CB), new bone (NB), reaction tissue (RT), membrane (M), and the yellow dashed line delimits the defect border (b). Images were acquired at an original magnification of 4× (scale bar: 2 mm), with higher-magnification details obtained at 20× (scale bar: 200 µm).

The most significant performance was observed in the photobiomodulation, fibrin biopolymer, biological membrane group (PFMG), which exhibited immature trabeculae extending toward the center of the defect, associated with denser collagen fibers and osteoid matrix formation (Figure 1). These interpretations are based on the qualitative histological observations and the quantitative histomorphometric findings presented in this section, and they support the hypothesis of a combined effect of PBM and membrane use in the early stage of repair [80,81,82,83], without implying direct causality. Moreover, the fibrin biopolymer used in this group rapidly polymerizes into a cohesive fibrin gel after the mixing of its components, functioning as a biodegradable scaffold that favors initial cell adhesion and matrix organization, which may contribute to the early repair pattern observed [48,49,53,54].

At the 42-day analysis, histological evaluation and histomorphometric assessment indicated that all groups showed signs of cortical remodeling and progressive replacement of connective tissue by bone tissue, but with distinct repair patterns. In the CG and FG, bone neoformation remained relatively restricted, presenting the lowest bone neoformation of the groups within this time frame, with a predominance of connective tissue in the central region of the defect, revealing a slower and more limited process. In the groups treated with membrane (CMG and FMG), more continuous trabeculae were observed, interspersed with areas of residual connective tissue, suggesting greater structural stability and organizational evolution of repair.

**Figure 2:**
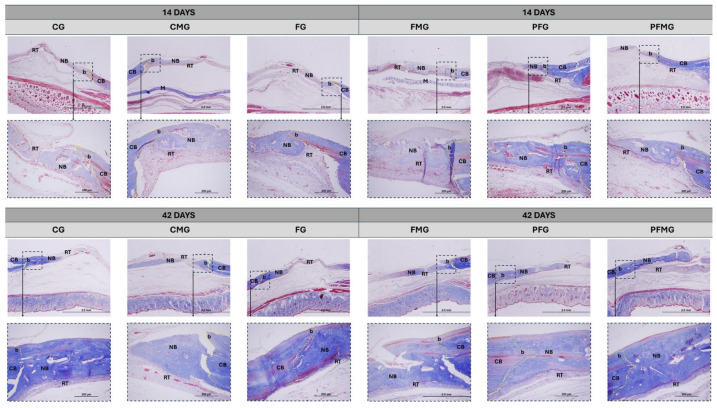
Masson’s trichrome–stained longitudinal sections of calvarial bone defects evaluated at 14 and 42 days. Control group (CG), fibrin biopolymer group (FG), clot and biological membrane group (CMG), fibrin biopolymer with biological membrane group (FMG), photobiomodulation with fibrin biopolymer group (PFG) and photobiomodulation, fibrin biopolymer, and biological membrane group (PFMG). Cortical bone (CB), new bone (NB), reaction tissue (RT), membrane (M), and the yellow dashed line delimits the defect border (b). Images were acquired at an original magnification of 4× (scale bar: 2 mm), with higher-magnification details obtained at 20× (scale bar: 200 µm).

In contrast, in the groups undergoing photobiomodulation (PFG and PFMG), more specifically the group that underwent the highest number of interventions/associated biomaterials (PFMG), a considerable increase in mineralized bone tissue was observed, consequently reducing the proportion of non-mineralized connective tissue. Masson’s trichrome staining (Figure 2) supported the identification of more mature collagen fibers and a greater extent of osteoid matrix, indicating a more advanced repair pattern in the groups that received PBM, with greater visualization in the PFMG [84,85,86]. In this context, the results observed at both experimental periods can be discussed in relation to previously reported effects of PBM and biomaterial-based approaches.

Consistently, the PFMG showed the most significant results, both at 14 and 42 days, combining the structural role of the membrane with the PBM protocol applied in this study. The presence of mature collagen fibers, associated with the formation of organized trabeculae, suggests a potential interaction between the two therapeutic resources. Previous studies report that PBM may be associated with changes in cellular and molecular events related to osteogenesis, such as osteoblastic activity, angiogenesis, and inflammatory modulation, which can be reflected in tissue maturation patterns [87,88,89]. The association with biomaterials, in turn, provides an osteoconductive framework, allowing the photobiomodulatory protocol to be applied in a stabilized defect microenvironment [90,91]. Taken together, these findings allow a broader interpretation of the combined regenerative strategy when compared with existing experimental models.

The findings suggest that the combination of heterologous fibrin biopolymer (CEVAP/UNESP Botucatu), GenDerm^®^ biological membrane (Baumer S.A., Mogi Mirim, São Paulo, Brazil), and PBM may be associated with improved bone repair patterns compared to the isolated use of each resource, based on the histological (Masson’s trichrome), histomorphometric, and birefringence outcomes reported. PBM was associated with modulation of the local biological response, with the potential to influence bone matrix deposition and collagen maturation, while the membrane acted to maintain space and protect the clot, important conditions for guided regeneration. Thus, the combined strategy was associated with a greater quantity and organization of newly formed bone tissue, approaching a more functional repair pattern. These results are consistent with previous studies reporting positive effects of integrating different therapeutic approaches in critical defects [92,93,94].

### 2.2. Collagen Fiber Birefringence Analysis

**Figure 3:**
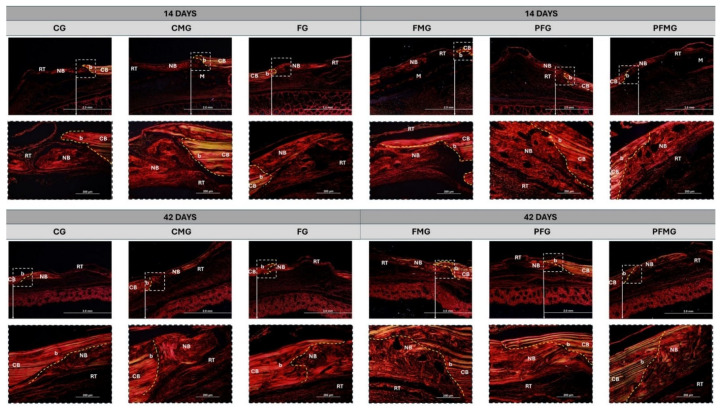
Picrosirius red–stained longitudinal sections of calvarial bone defects analyzed under polarized light at 14 and 42 days. Control group (CG), fibrin biopolymer group (FG), clot and biological membrane group (CMG), fibrin biopolymer with biological membrane group (FMG), photobiomodulation with fibrin biopolymer group (PFG) and photobiomodulation, fibrin biopolymer, and biological membrane group (PFMG). The yellow dashed line delimits the defect edge (b). Membrane (M), cortical bone (CB), new bone (NB), reaction tissue (RT). Images were acquired at an original magnification of 4× (scale bar: 2 mm), with higher-magnification details obtained at 20× (scale bar: 200 µm).

Analysis under polarized light with Picrosirius red staining, performed as described in the Materials and Methods, allowed evaluation of the organization and degree of maturation of collagen fibers throughout bone repair. At 14 days, all groups showed a predominance of thin, disorganized fibers, characteristic of type III collagen, indicating an early stage of tissue formation. However, in the groups undergoing photobiomodulation (PFG and PFMG), polarized light microscopy revealed areas of transition to thicker, yellowish fibers, suggesting the onset of collagen maturation toward type I (Figure 3). The following interpretation is based on the birefringence patterns observed and their comparison with previously published studies. These findings, derived from birefringence analysis, support the possibility that PBM may be associated with earlier collagen organization and matrix maturation, in agreement with studies demonstrating modulation of collagen organization and mineralized matrix deposition under PBM protocols [95,96,97].

Over a period of 42 days, birefringence analysis demonstrated a predominance of thick collagen fibers oriented parallel in virtually all groups, despite presenting in different concentrations, with a predominance of the characteristic pattern of type I collagen (Figure 3). This finding indicates the progressive replacement of the initial connective tissue by a more mature and organized matrix. It should be noted, however, that the groups treated with photobiomodulation, especially PFMG, exhibited greater fiber density and organization, as observed under polarized light microscopy, as well as a more homogeneous transition between the margins of the remaining bone and the central region of the defect. These observations align with previous findings reported by Della Colleta et al. [85], indicating that laser-based therapies are linked to enhanced collagen organization and bone formation, thereby reinforcing their potential role as adjunctive approaches in regenerative strategies. From a comparative perspective, these results can be discussed in the context of combined regenerative approaches described in the literature.

In addition, the combined effect of fibrin biopolymer and biological membrane, associated with PBM, resulted in a more advanced repair pattern as assessed by birefringence outcomes. This potential interaction can be interpreted considering the joint action of the biological matrix, which promotes cell adhesion and extracellular matrix deposition, together with the PBM protocol, which may modulate cellular processes and angiogenesis [98,99,100,101]. Thus, the earlier and more organized maturation of collagen tissue in the PFG and PFMG suggests a potential pathway for optimizing guided bone regeneration, especially in clinical situations where the goal is to reduce repair time and improve the quality of the tissue formed. This effect may be partially attributed to the fibrin biopolymer, which rapidly polymerizes into a cohesive fibrin gel acting as a biodegradable scaffold that supports initial cell adhesion and matrix organization, thereby contributing to a more favorable early repair environment [53,54].

### 2.3. Histomorphometric and Statistical Analysis

At 14 days, histomorphometric analysis, specifically the quantitative assessment of hematoxylin–eosin–stained slides as described in the Materials and Methods showed clear differences between the experimental groups. Based on histomorphometric analysis, the PFMG presented the highest mean bone formation value (55.88 ± 2.88), which differed statistically from the other experimental groups, including CG (40.55 ± 11.25), CMG (40.71 ± 6.33), FG (36.97 ± 5.15), FMG (42.22 ± 7.85), and particularly PFG (30.86 ± 6.52), which showed the lowest mean value at this time point (Table 1). Based on the quantitative data obtained, the following considerations place the results within a broader experimental context. This finding supports a potential interaction between the presence of a biological membrane and the PBM protocol in the early stage of repair, based on quantitative outcomes.

At 42 days, histomorphometric evaluation indicated an overall increase in bone repair when compared with the earlier time point, reflecting the expected temporal progression of healing in critical-size defects. The CMG (69.22 ± 3.92) and PFMG (69.49 ± 6.73) groups had the highest bone neoformation values, statistically superior (*p* < 0.05) to the CG (47.13 ± 3.92), FG (56.42 ± 7.31), FMG (51.68 ± 7.78), and PFG (46.10 ± 5.82) groups, indicating that the presence of the biological membrane played a fundamental role in maintaining space and promoting osteoconduction over time (Table 1). These findings are supported by the quantitative histomorphometric data and are consistent with the qualitative observations in histological sections stained with Masson’s trichrome. The superiority of the PFMG over the others reinforces the therapeutic potential of combining regenerative resources, corroborating studies that highlight the role of PBM in stimulating osteoblastic activity and modulating the inflammatory microenvironment [102,103,104].

#### 2.3.1. 14 Days

In the membrane groups, regardless of the use of fibrin biopolymer or photobiomodulation (CMG and PFMG), greater connective tissue organization was observed compared to the control group, based on the histological sections evaluated. However, the deposition of new mineralized tissue remained limited to the margins of the defect, suggesting that the role of the membrane in this initial period was more to stabilize the clot and maintain the space than to directly induce bone neoformation. The descriptive observations obtained at this time point can be interpreted in light of previous experimental findings. These results corroborate findings previously reported by other authors, who describe that membranes act as physical barriers that favor the repair microenvironment, but whose total effectiveness depends on association with additional stimuli, such as PBM [105,106].

**Figure 4:**
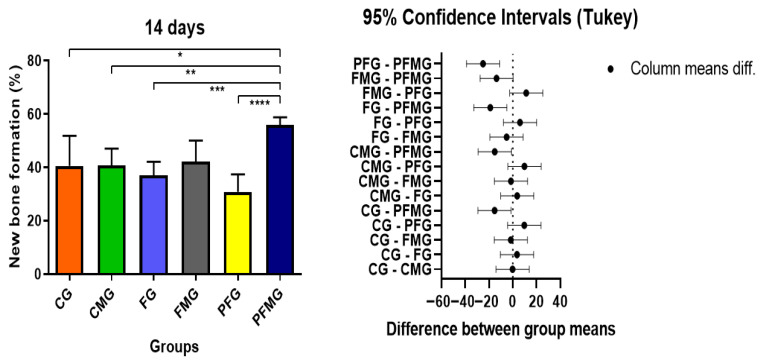
Comparison of bone formation among experimental groups at the 14-day time point. Asterisks (*, **, ***, or ****) indicate statistically significant differences between groups as determined by one-way ANOVA followed by Tukey’s post hoc test (*p* < 0.05). The PFMG presented higher bone formation values compared with the CG, CMG, FG, FMG, and PFG, based on the statistical analysis performed.

The analysis indicated that PBM may have positively influenced bone regeneration. Although the group irradiated without a membrane (PFG) had the lowest bone formation values, the PFG and PFMG showed a greater presence of immature bone trabeculae, which extended toward the central region of the defect, as observed in the histological sections and supported by the histomorphometric data. These observations indicate that PBM may be associated with changes in the local repair environment, including inflammatory modulation, angiogenic responses, and osteoprogenitor cell activity, as reported in studies describing increased expression of osteogenic markers in PBM-treated tissues [90,107,108,109,110]. The data referring to new bone formation at 14 days highlight the differences between the groups and illustrate the potential additive effect of the combination of membrane and PBM in the early stages of repair (Figure 4). Considering these observations, the early repair outcomes can be discussed in comparison with similar experimental models.

Overall, the results obtained at 14 days indicate that the combination of membrane and PBM may have provided significant advantages in the initial repair of critical defects, as seen in other experimental studies [111,112,113]. However, residual connective tissue in all groups indicates that bone maturation was still in its early stages, this finding highlights the importance of monitoring the progression of the repair process over longer periods, especially at 42 days, when a higher degree of trabecular maturation, collagen reorganization, and consolidation of the preliminary findings described in this initial phase are expected [106,114].

#### 2.3.2. 42 Days

**Figure 5:**
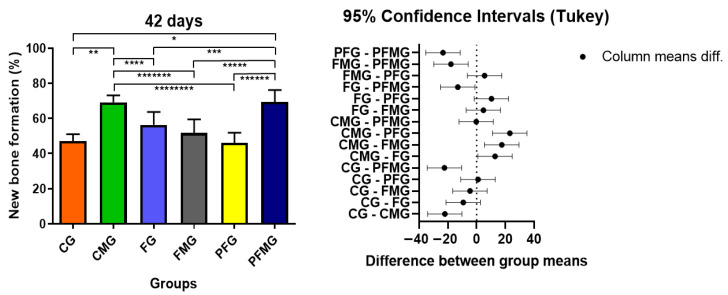
Bone formation assessed at 42 days postoperatively across the experimental groups. Asterisks (*, **, ***, ****, *****, ******, ******* or ********) denote statistically significant differences identified by one-way ANOVA followed by Tukey’s post hoc test (*p* < 0.05). Based on the statistical analysis, the CMG and PFMG exhibited higher bone formation values compared with the CG and PFG at this time point.

At 42 days, the results confirmed and reinforced the trends observed in the initial period. Both CMG and PFMG demonstrated significantly greater bone neoformation compared with CG and PFG (*p* < 0.05), emphasizing the role of the biological membrane and its association with PBM in the later stages of healing. These findings graphically illustrate the bone formation values for all experimental groups, highlighting the superior performance of those treated with membrane and PBM (Figure 5). These quantitative findings allow comparison with previous reports on guided bone regeneration strategies. These results corroborate evidence that the use of barrier membranes in guided bone regeneration protocols favors osteoconduction and repair organization, with reported improvements when associated with PBM protocols [84,88,102].

Histomorphometric analysis also demonstrated that PBM was associated with patterns consistent with increased bone tissue maturation. In the groups treated with laser (PFG and PFMG), greater mineralized matrix deposition was observed in the histological sections, a finding consistent with experimental PBM studies reporting modulation of cell differentiation and expression of osteogenic markers [94,99,100].

Furthermore, data suggest that photobiomodulation may contribute to a more balanced regenerative microenvironment by modulating the inflammatory response. Evidence from prior investigations indicates that PBM has been associated with shifts in the inflammatory profile, characterized by reduced levels of pro-inflammatory mediators and increased expression of anti-inflammatory cytokines, which may influence the quality of tissue repair [62,100,101]. In the present study, this pattern was particularly evident in the PFMG, which presented the highest degree of trabecular organization, corroborating previous studies that described the association between biomaterials and PBM in reducing inflammation and promoting bone maturation [89,115]. Finally, additional aspects related to the PBM protocol can be discussed based on the observed outcomes and existing evidence.

Another relevant point is the confirmation that the energy dose used (6 J·cm^−2^) is within the so-called “therapeutic window” of PBM, described as between 0.05 and 10 J·cm^−2^, avoiding the bioinhibitory effects observed at higher doses [116]. This dosage adjustment may explain the consistent response observed in the stimulated groups. Furthermore, analysis using specific staining and birefringence outcomes showed thicker and well-organized collagen fibers in the groups undergoing PBM, suggesting facilitation of the transition from type III to type I collagen, an aspect already discussed in the literature as a marker of advanced bone maturation [89].

## 3. Conclusions

The present study evaluated the effects of photobiomodulation on critical calvarial bone defects in rats filled with different combinations of biomaterials, including heterologous fibrin biopolymer and bovine cortical bone biological membrane. The use of histomorphometric analyses enabled effective characterization of the bone response, allowing comparison of the performance of the different therapeutic approaches. In general, PBM was associated with greater mineralized matrix deposition and better tissue organization, especially when combined with biomaterials. The groups that received the combination of biological membrane and fibrin biopolymer showed signs of more advanced repair, suggesting that the combined use of these strategies may contribute to a microenvironment more favorable to bone neoformation. Moreover, the fibrin biopolymer’s ability to polymerize into a stable, biodegradable gel with intrinsic scaffold and bioadhesive properties may also support early phases of bone repair identified in this study.

However, despite the positive trend observed, the regenerative process was not complete in any of the groups, which reinforces the need for cautious interpretation of these results. Taken together, these observations suggest that PBM may be associated with supportive effects in guided bone regeneration approaches when combined with biomaterials; however, additional investigations are required to further clarify the magnitude and reproducibility of these outcomes across different experimental settings. Thus, we recommend continuing preclinical investigations with varying irradiation parameters, types of biomaterials, and observation times, as well as future controlled clinical trials to validate the translational applicability and confirm the real impact of this association in the clinical practice of tissue engineering and bone regeneration.

From a translational perspective, the present findings contribute to a better understanding of how combined regenerative strategies may influence bone repair in critical-size defects. Although this is a preclinical study, the observed associations between photobiomodulation, fibrin-based scaffolding, and barrier membrane use support further investigation of integrated approaches aimed at optimizing guided bone regeneration protocols. These results may help inform future experimental designs and the development of more effective regenerative strategies for oral and maxillofacial bone reconstruction, while reinforcing the need for additional preclinical and clinical studies to validate their applicability.

## 4. Materials and Methods

### 4.1. Ethical Aspects

All experimental procedures were performed in compliance with the ethical principles of the Declaration of Helsinki and were approved by the Ethics Committee on Animal Use (CEUA) of the University of Marília under protocols 04/2018 and 06/2018 (CIAEP-01.0218.2014). All experimental procedures followed the ARRIVE guidelines and the principles of the NC3Rs, ensuring animal welfare throughout the study. Animals were monitored daily for general health status and signs of pain or distress, and no adverse events or complications requiring animal exclusion were observed.

### 4.2. Selection and Maintenance of the Animals

This study utilized sixty adult male Wistar rats (*Rattus norvegicus*), approximately 90 days old, with an average body weight of 250 g. Animals were obtained from ANILAB (Paulínia, Brazil) and housed at the Central Animal Facility of the University of Marília, where all experimental procedures were conducted.

Male rats were selected due to the influence of sex hormones on bone metabolism, particularly the inhibitory effect of estrogen on periosteal bone formation, which could interfere with the outcomes in females [117,118].

The sample size was defined based on previous study to ensure adequate statistical power while complying with bioethical principles [119].

Animals were housed in standard laboratory conditions, in conventional cages with ad libitum access to food and water, under controlled temperature and a 12 h light/dark cycle.

### 4.3. Randomization of Experimental Groups and Materials Used

**Figure 6:**
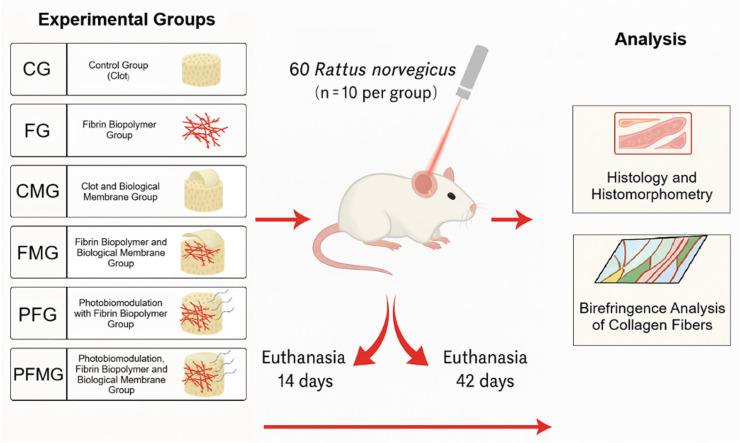
Sixty adult male *Rattus norvegicus* were randomly distributed into six experimental groups (*n* = 10 per group): control group (CG), fibrin biopolymer group (FG), clot plus biological membrane group (CMG), fibrin biopolymer plus biological membrane group (FMG), photobiomodulation with fibrin biopolymer group (PFG), and photobiomodulation associated with fibrin biopolymer and biological membrane group (PFMG). Photobiomodulation was applied according to the protocol described in the Materials and Methods, following wound closure. Animals were euthanized at 14 and 42 days for histological, histomorphometric, and collagen birefringence analyses. Colors are used for illustrative purposes only to facilitate visualization of the experimental design.

The animals were randomly allocated into six experimental groups based on the type of material used to fill the critical bone defect and the application of the photobiomodulation protocol: Control group (Clot) (CG; *n* = 10), fibrin biopolymer group (FG; *n* = 10), clot and biological membrane group (CMG; *n* = 10), fibrin biopolymer, biological membrane group (FMG; *n* = 10), photobiomodulation with fibrin biopolymer group (PFG; *n* = 10), and photobiomodulation, fibrin biopolymer and biological membrane group (PFMG; *n* = 10) (Figure 6 and Table 2).

### 4.4. Heterologous Fibrin Biopolymer

The heterologous fibrin biopolymer used in this study was supplied by the Center for the Study of Venoms and Venomous Animals (CEVAP, São Paulo State University—UNESP, Botucatu, Brazil), with its composition and formulation detailed in patent BR 102014011432-7, granted by the National Institute of Industrial Property (INPI) on 6 July 2022. The material is composed of three separate fractions that were stored under frozen conditions and thawed immediately prior to application. Prior to application, all fractions were gently homogenized under sterile conditions, following standardized handling procedures to preserve their biological activity, as previously described for this heterologous fibrin system [120].

Fraction 1 is composed of a thrombin-like serine protease (gyroxin) isolated from the venom of *Crotalus durissus terrificus* [51,52], Fraction 2 consists of a calcium chloride diluent, and Fraction 3 corresponds to a fibrinogen-rich cryoprecipitate obtained from *Bubalus bubalis* blood [49,56]. For preparation, the fractions were mixed sequentially immediately before use, using fixed volumes of 20 µL of Fraction 1, 20 µL of diluent (Fraction 2), and 40 µL of Fraction 3. Upon mixing, rapid polymerization occurred within a short period, resulting in the formation of a cohesive and translucent fibrin gel that mimics the final step of the coagulation cascade and produces a dense fibrin network, as consistently reported for this biopolymer formulation [120,121].

The fibrin biopolymer was applied in a single step to completely fill the critical-size defect. Care was taken to avoid overflow beyond the defect margins. Polymerization occurred rapidly within the defect after mixing the fractions, resulting in a cohesive gel. In groups receiving the biological membrane, the membrane was positioned after in situ polymerization of the fibrin biopolymer, covering the defect without exerting pressure on the underlying tissue.

The resulting gel behaves as a biodegradable scaffold capable of promoting cell adhesion, supporting tissue organization, and providing intrinsic hemostatic and bioadhesive properties [53,55]. In addition, due to its ability to incorporate and gradually release bioactive molecules, this fibrin biopolymer and other similar fibrin-based and biomimetic scaffolds have been increasingly investigated for applications in nerve repair, skin healing, bone regeneration, periodontal procedures, and drug-delivery strategies [122,123,124,125]. These characteristics justify its expanding interest in experimental and translational research, as well as the growing investigation of comparable fibrin-based systems with analogous biological and physicochemical properties [126,127,128,129].

### 4.5. Bovine Bone Cortical Biological Membrane

The membrane used for GBR was GenDerm^®^ (Baumer, Mogi Mirim, Brazil), obtained from bovine cortical bone and characterized by its flexibility and resorption capacity. The production process yields a membrane characterized by a porous and acellular architecture, high biocompatibility, and absence of antigenic or pyrogenic components, with stringent purification steps ensuring freedom from heavy metal contamination and residual proteins. For this experiment, the 20 × 20 mm version was used, manually adjusted to fit the bone defect created in the experimental model.

In the CMG group animals, the membrane was positioned between the clot filling the bone cavity and the periosteum. For the FMG and PFMG, it was inserted between the cavity filled with fibrin biopolymer gel and the periosteum. The membrane was cut to a diameter of 7.0 mm to ensure peripheral sealing of the defect and prevent the migration of epithelial cells into the cavity, thus avoiding interference by these cells in the bone repair and healing process.

### 4.6. Experimental Surgery

For the experimental surgery, animals were subjected to general anesthesia by intramuscular injection of tiletamine hydrochloride associated with zolazepam hydrochloride (10 mg/kg—Telazol^®^; Fort Dodge Laboratories, Fort Dodge, IA, USA), under veterinary supervision. After trichotomy and antisepsis of the fronto-parietal region, a semilunar incision was performed, followed by periosteal detachment to expose the parietal bones.

A standardized circular critical-size defect (5.0 mm in diameter) was created in the center of the parietal bones using a trephine drill at low speed (Neodent^®^, Curitiba, Brazil) under continuous sterile saline irrigation, preserving the dura mater and underlying brain tissue. In the FMG and PFMG, the defects were filled with heterologous fibrin biopolymer and covered with a biological membrane, both carefully positioned without exerting pressure on adjacent tissues.

After defect filling, the periosteum and soft tissues were repositioned, and the integument was sutured with simple interrupted sutures using 4-0 silk thread (Ethicon^®^, Johnson & Johnson, Sao Paulo, Brazil). The surgical site was disinfected using sterile gauze moistened with a 2% chlorhexidine antiseptic solution (Riohex^®^, Rioquímica Ltda., São José do Rio Preto, Brazil).

Postoperative recovery occurred under controlled conditions until full anesthetic recovery. Antibiotic prophylaxis was administered in a single intramuscular dose with Flotril^®^ 2.5% (Schering-Plough, Rio de Janeiro, Brazil) 0.2 mL/kg, and initial analgesia with Dipyrone Analgex V^®^ (Agener União, Sao Paulo, Brazil) at a dose of 0.06 mL/kg. Analgesia was maintained for three days, associated with the continuous use of Paracetamol (Generic Medicine, Medley, Sao Paulo, Brazil) diluted in the water from the drinkers available to the animals (200 mg/kg, 6 drops/animal) until the moment of euthanasia.

Throughout the experimental period, animals were monitored daily for general health status and signs of pain or discomfort, in accordance with animal welfare and ARRIVE guidelines [125].

### 4.7. Protocol for Photobiomodulation Therapy (PBM)

Animals allocated to the PFG and PFMG received photobiomodulation therapy using a GaAlAs (Gallium–Aluminum–Arsenide) laser device (Laserpulse^®^, Ibramed, Amparo, Brazil). During irradiation, the animals were gently manually restrained to allow adequate exposure of the cranial region, and no additional anesthesia was required for the procedure.

**Figure 7:**
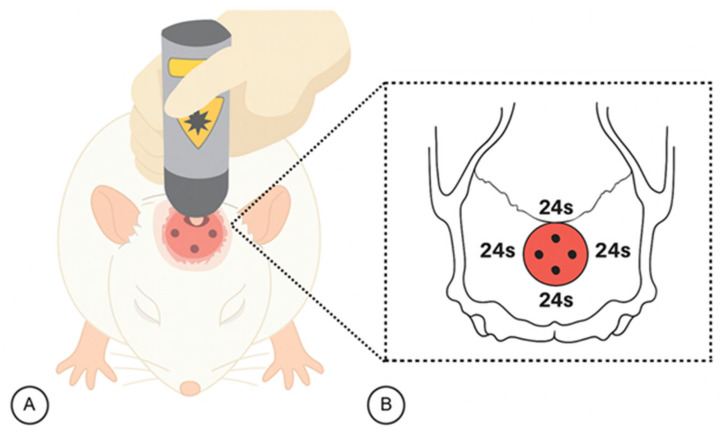
Illustration of the established photobiomodulation protocol, considering time and positioning. (**A**) Positioning of the laser emitter, kept in contact with the animal’s skin at 90°. (**B**) Illustration referring to the 4 cross-shaped points on the surgical site that received the laser.

The photobiomodulation protocol employed continuous-wave emission at a wavelength of 830 nm, with an output power of 30 mW and an energy density of 6 J/cm^2^. Irradiation was delivered for 24 s at four equidistant points arranged in a cross configuration over the surgical area, corresponding to a beam area of 0.116 cm^2^ and a power density of 258.6 mW/cm^2^. Throughout the application, the emitter was maintained perpendicular (90°) to the skin surface, resulting in a total irradiation time of 96 s per session (Figure 7 and Table 3).

Treatment was started immediately after surgery, after wound closure, and repeated three times a week until the animals were euthanized. The laser emissions were calibrated beforehand on the equipment itself, ensuring the standardization of the energy released. This protocol follows the same one used by de Oliveira Gonçalves et al. [58], serving as a comparative basis for the present investigation.

### 4.8. Surgical Procedure for Tissue Extraction

After the experimental periods of 14 and 42 days, five animals from each group (CG, CMG, FG, FMG, PFG, and PFMG) were weighed and euthanized. The entire process was carried out in a calm and isolated environment to avoid stress on the other animals. Euthanasia was performed by administering 2.5% Sodium Thiopental (150 mg/kg, Thiopental^®^, Cristalia, Itapira, Brazil) intraperitoneally in the lower left abdominal quadrant, combined with Lidocaine (10 mg/kg) for local analgesia.

Following confirmation of the absence of vital reflexes, the cranial segment containing the defect was carefully excised using a conical carbide bur mounted on a low-speed handpiece (Dabi Atlante^®^, Ribeirão Preto, Brazil), under continuous irrigation to preserve the supraperiosteal soft tissues. The collected specimens were immediately immersed in 10% buffered formalin (pH 7.2) for a fixation period of one week before being forwarded for histological processing.

Finally, the animals were placed in disposal bags suitable for biological waste, kept frozen, and subsequently disposed of according to current biosafety standards.

### 4.9. Histotechnical Processing

The specimens were initially rinsed under running water for 24 h and subsequently subjected to demineralization using an ethylenediaminetetraacetic acid (EDTA) solution. The decalcifying solution was prepared with 4.13% Tritriplex^®^ III (Merck KGaA, Darmstadt, Hessen, Germany) and 0.44% sodium hydroxide (Labsynth, São Paulo, Brazil) and was renewed weekly over a period of approximately 60 days. The progression of the decalcification process was periodically assessed through radiographic examination using In-sight adult IP-21 F-Speed periapical films (Carestream^®^, New York, NY, USA).

After completion of demineralization, the samples were dehydrated through a graded ethanol series, followed by clearing in xylene. The specimens were then embedded in Histosec^®^ paraffin (Merck, Hessen, Germany) in accordance with the standardized histological processing protocol adopted by the Anatomy Research Laboratory of the Bauru School of Dentistry (FOB-USP).

### 4.10. Histomorphometric Analysis of Defects Stained with Hematoxylin-Eosin and Masson’s Trichrome

For the histomorphometric analysis of the areas corresponding to bone defects, the extent of each defect in the different experimental groups was fully considered in order to characterize the tissue repair pattern in each condition. Sections were stained with hematoxylin and eosin to enable assessment of granulation tissue, identification of bone tissue presence and maturation stage (immature or lamellar), and evaluation of defect filling by newly formed matrix. In addition, Masson’s trichrome staining was employed to enhance visualization of collagen fiber deposition and organization, as well as to distinguish connective tissue areas from regions undergoing bone formation, thereby allowing a more comprehensive assessment of the regenerative process.

For each defect, four semi-serial sections were examined using an Olympus^®^ BX50 light microscope (Olympus Corporation, Tokyo, Japan). Image acquisition was performed at magnifications of 4× and 20× with an attached Olympus DP71 digital camera, operated through DP Controller software (version 3.2.1.276), generating images with a resolution of 1360 × 1024 pixels and a spot setting of 1%. All microscopic analyses were conducted at the Anatomy Research Laboratory of the Bauru School of Dentistry (FOB-USP).

The captured images were processed and quantified using the Aperio ImageScope system (Leica Biosystems Imaging, Inc., version 12.4.6.5003, Wetzlar, Germany). Measurements were obtained for three main parameters: bone formation area, percentage of remaining biomaterial, and proportion of connective tissue present within the defect.

### 4.11. Analysis of the Birefringence of Collagen Content in Bone Defects

Sections stained with Picrosirius Red were examined under polarized light using a Leica^®^ DM IRB/E inverted microscope (Leica Microsystems®, Wetzlar, Germany) equipped with a polarizing filter, at the Integrated Research and Innovation Center of the Bauru School of Dentistry (CIPI/FOB/USP). Analyses were conducted at magnifications of 4× and 20× to allow qualitative and quantitative assessment of the newly formed organic matrix during the experimental healing periods.

For each captured image, the RGB color profile was analyzed, and the area or percentage corresponding to each collagen fiber type was calculated. Bone tissue was identified based on its irregular and disorganized fibrillar arrangement, displaying birefringence colors ranging from orange-red, indicative of less organized primary bone, to yellowish-green, associated with more mature lamellar bone, depending on fiber thickness. To ensure consistency across analyses, the intensity and orientation of polarized light were standardized, with the polarizing filter positioned at 90° relative to the light source. Quantitative analysis of birefringence intensity was performed using KS 300/400 AxioVision software (version 4.8, Carl Zeiss, Jena, Germany).

### 4.12. Statistical Analysis

Statistical analyses were conducted for all experimental groups at both evaluation periods, taking into account the central region of the defect, cortical bone discontinuity, and the area of newly formed bone. Histomorphometric data were initially assessed for normality using the Kolmogorov–Smirnov test and for homogeneity of variances using Bartlett’s test. As the data met parametric assumptions, intergroup comparisons were performed using one-way analysis of variance (ANOVA), followed by Tukey’s post hoc test, with the level of significance set at *p* < 0.05.

One-way ANOVA followed by Tukey’s post hoc test was applied for inter-group comparisons within the same experimental period, whereas the unpaired *t*-test was used for specific pairwise comparisons when appropriate.

In addition, intra-period comparisons between specific groups were carried out using the unpaired Student’s *t*-test, also adopting a significance threshold of *p* < 0.05. All statistical analyses and graphical representations were generated using GraphPad Prism^®^ software, version 8 (GraphPad Software, La Jolla, CA, USA).

## Figures and Tables

**Table 1:** Mean values and standard deviations (%) of bone formation in the experimental groups CG, CMG, FG, FMG, PFG, and PFMG, evaluated at 14 and 42 days.

PERIOD	CG	CMG	FG	FMG	PFG	PFMG
14 days	40.55 ± 11.25 Bb	40.71 ± 6.33 Bb	36.97 ± 5.15 Bb	42.22 ± 7.85 Bb	30.86 ± 6.52 Bb	55.88 ± 2.88 Ba
42 days	47.13 ± 3.92 Ab	69.22 ± 3.92 Aa	56.42 ± 7.31 Ab	51.68 ± 7.78 Ab	46.10 ± 5.82 Ab	69.49 ± 6.73 Aa

Data are presented as mean ± standard deviation. Distinct uppercase letters (A ≠ B) denote statistically significant differences between experimental periods within the same group (14 vs. 42 days), whereas distinct lowercase letters (a ≠ b) indicate significant differences among groups evaluated at the same time point. Statistical comparisons between periods were performed using Student’s *t*-test, while intergroup comparisons were conducted using Tukey’s post hoc test, with the significance threshold set at *p* < 0.05.

**Table 2:** Summary of experimental groups and regenerative components used in the study.

Experimental Groups	Blood Clot	Fibrin Biopolymer	Biological Membrane	Photobiomodulation
CG (Control group)	Yes	No	No	No
FG (Fibrin group)	No	Yes	No	No
CMG (Clot + membrane group)	Yes	No	Yes	No
FMG (Fibrin + membrane group)	No	Yes	Yes	No
PFG (PBM + fibrin group)	No	Yes	No	Yes
PFMG (PBM + fibrin + membrane group)	No	Yes	Yes	Yes

Control group (CG), fibrin biopolymer group (FG), clot and biological membrane group (CMG), fibrin biopolymer with biological membrane group (FMG), photobiomodulation with fibrin biopolymer group (PFG), photobiomodulation, fibrin biopolymer, and biological membrane group (PFMG), and photobiomodulation (PBM).

**Table 3:** Photobiomodulation (PBM) parameters used in the study.

Parameters	Specification
Light source	Low-level laser (GaAlAs)
Wavelength (nm)	830 nm (near-infrared)
Emission mode	Continuous wave
Output power (mW)	30 mW
Power density/Irradiance (mW/cm^2^)	258.6 mW/cm^2^
Energy density/Fluence (J/cm^2^)	6 J/cm^2^
Spot size/Beam area (cm^2^)	0.116 cm^2^
Application time per point (s)	24 s
Number of irradiation points	4

Photobiomodulation (PBM), near-infrared (NIR).

## Data Availability

Data presented in this study are available on request from the corresponding author.

## Abbreviations

The following abbreviations are used in this manuscript:

CG	Control group
CMG	Clot and membrane group
FG	Fibrin biopolymer group
FMG	Fibrin biopolymer and membrane group
PFG	Photobiomodulation and fibrin biopolymer group
PFMG	Photobiomodulation, fibrin biopolymer and membrane group
GBR	Guided bone regeneration
GaAlAs	Aluminum gallium arsenide diode
EDTA	Ethylenediamine tetraacetic acid
M	Membrane
CB	Cortical bone
RT	Reaction tissue
b	Defect border
PBM	Photobiomodulation
LLLT	Low-level laser therapy

## References

[1] Buck D.W., Dumanian G.A. (2012). Bone Biology and Physiology. Plast. Reconstr. Surg..

[2] Iaquinta M.R., Montesi M., Mazzoni E. (2024). Advances in Bone Biology. Int. J. Mol. Sci..

[3] Lecka-Czernik B., Rosen C.J., Napoli N. (2025). The Role of Bone in Whole-Body Energy Metabolism. Nat. Rev. Endocrinol..

[4] Lafage-Proust M.-H., Magne D. (2024). Biology of Bone Mineralization and Ectopic Calcifications: The Same Actors for Different Plays. Arch. De Pédiatrie.

[5] Fanti P., Monier-Faugere M.-C., Geng Z., Cohen D., Malluche H.H. (1997). Moderately High Consumption of Ethanol Suppresses Bone Resorption in Ovariectomized but Not in Sexually Intact Adult Female Rats. Alcohol. Clin. Exp. Res..

[6] Fazzalari N.L. (2011). Bone Fracture and Bone Fracture Repair. Osteoporos. Int..

[7] Kirk B., Lombardi G., Duque G. (2025). Bone and Muscle Crosstalk in Ageing and Disease. Nat. Rev. Endocrinol..

[8] Wherry S.J. (2024). Calcium Balance: Considerations for the Bone Response to Exercise. J. Bone Miner. Res..

[9] Nowak A., Ogurkowska M. (2024). Bone Health and Physical Activity—The Complex Mechanism. Aging Dis..

[10] Nogueira D.M.B., Figadoli A.L.d.F., Alcantara P.L., Pomini K.T., Santos German I.J., Reis C.H.B., Rosa Júnior G.M., Rosso M.P.d.O., Santos P.S.d.S., Zangrando M.S.R. (2022). Biological Behavior of Xenogenic Scaffolds in Alcohol-Induced Rats: Histomorphometric and Picrosirius Red Staining Analysis. Polymers.

[11] Santos German I.J., Pomini K.T., Bighetti A.C.C., Andreo J.C., Reis C.H.B., Shinohara A.L., Rosa Júnior G.M., Teixeira D.d.B., Rosso M.P.d.O., Buchaim D.V. (2020). Evaluation of the Use of an Inorganic Bone Matrix in the Repair of Bone Defects in Rats Submitted to Experimental Alcoholism. Materials.

[12] Giannoudis P.V., Einhorn T.A., Marsh D. (2007). Fracture Healing: The Diamond Concept. Injury.

[13] Győri D.S. (2024). Research on Bone Cells in Health and Disease. Int. J. Mol. Sci..

[14] Lau C.S., Park S.Y., Ethiraj L.P., Singh P., Raj G., Quek J., Prasadh S., Choo Y., Goh B.T. (2024). Role of Adipose-Derived Mesenchymal Stem Cells in Bone Regeneration. Int. J. Mol. Sci..

[15] Łuczak J.W., Palusińska M., Matak D., Pietrzak D., Nakielski P., Lewicki S., Grodzik M., Szymański Ł. (2024). The Future of Bone Repair: Emerging Technologies and Biomaterials in Bone Regeneration. Int. J. Mol. Sci..

[16] Anwar A., Kaur T., Chaugule S., Yang Y.-S., Mago A., Shim J.-H., John A.A. (2024). Sensors in Bone: Technologies, Applications, and Future Directions. Sensors.

[17] Sadeghi S., Pezeshgi S., Sadeghi R., Bayan N., Farrokhpour H., Amanollahi M., Bereimipour A., Abolghasemi Mahani A. (2024). Clinical Application of Biomaterials in Orbital Implants: A Systematic Review. Int. Ophthalmol..

[18] Trevisiol C.H., Turner R.T., Pfaff J.E., Hunter J.C., Menagh P.J., Hardin K., Ho E., Iwaniec U.T. (2007). Impaired Osteoinduction in a Rat Model for Chronic Alcohol Abuse. Bone.

[19] Buchaim R.L., Andreo J.C., Rodrigues A.C., Buchaim D.V., Dias D.V., Daré L.R., Roque D.D., Roque J.S. (2013). The Action of Demineralized Bovine Bone Matrix on Bone Neoformation in Rats Submitted to Experimental Alcoholism. Arq. Bras. Med. Vet. Zootec..

[20] Rosso M.P.d.O., Oyadomari A.T., Pomini K.T., Della Coletta B.B., Shindo J.V.T.C., Ferreira Júnior R.S., Barraviera B., Cassaro C.V., Buchaim D.V., Teixeira D.d.B. (2020). Photobiomodulation Therapy Associated with Heterologous Fibrin Biopolymer and Bovine Bone Matrix Helps to Reconstruct Long Bones. Biomolecules.

[21] Nogueira D.M.B., Rosso M.P.d.O., Buchaim D.V., Zangrando M.S.R., Buchaim R.L. (2024). Update on the Use of 45S5 Bioactive Glass in the Treatment of Bone Defects in Regenerative Medicine. World J. Orthop..

[22] Jakob H., Campbell C.D., Stemberger A., Wriedt-Lübbe I., Blümel G., Replogle R.L. (1984). Combined Application of Heterologous Collagen and Fibrin Sealant for Liver Injuries. J. Surg. Res..

[23] Miron R.J. (2024). Optimized Bone Grafting. Periodontol. 2000.

[24] Moraschini V., Louro R.S., Son A., Calasans-Maia M.D., Sartoretto S.C., Shibli J.A. (2024). Long-term Survival and Success Rate of Dental Implants Placed in Reconstructed Areas with Extraoral Autogenous Bone Grafts: A Systematic Review and Meta-analysis. Clin. Implant. Dent. Relat. Res..

[25] Tommasato G., Del Fabbro M., Oliva N., Khijmatgar S., Grusovin M.G., Sculean A., Canullo L. (2024). Autogenous Graft versus Collagen Matrices for Peri-Implant Soft Tissue Augmentation. A Systematic Review and Network Meta-Analysis. Clin. Oral Investig..

[26] Develioglu H., Saraydın S., Kartal Ü., Taner L. (2010). Evaluation of the Long-Term Results of Rat Cranial Bone Repair Using a Particular Xenograft. J. Oral Implantol..

[27] Ladd R., Demer A., Ochoa S., Arpey C. (2024). Bovine Collagen Xenografts as Cost-Effective Adjuncts for Granulating Surgical Defects. Dermatol. Surg..

[28] Qasim S.S.B., Trajkovski B., Zafiropoulos G.-G. (2024). The Response of Human Osteoblasts on Bovine Xenografts with and without Hyaluronate Used in Bone Augmentation. J. Biomater. Sci. Polym. Ed..

[29] Wang K., Zhang J., Ding M., Xie Y., Wang Y., Jin C., Yan M., Liu L., Ding C., Chen X. (2025). Comparative Evaluation of Porcine and Bovine Bone Xenografts in Bone Grafting: A Systematic Review and Meta-Analysis. Int. J. Implant. Dent..

[30] Wang H.L., Carroll M.J. (2001). Guided Bone Regeneration Using Bone Grafts and Collagen Membranes. Quintessence Int..

[31] Ashfaq R., Kovács A., Berkó S., Budai-Szűcs M. (2024). Developments in Alloplastic Bone Grafts and Barrier Membrane Biomaterials for Periodontal Guided Tissue and Bone Regeneration Therapy. Int. J. Mol. Sci..

[32] Liao C.D., Rodriguez E., Zhao K., Kunda N., George F. (2023). Complications Following Alloplastic Chin Augmentation. Ann. Plast. Surg..

[33] Carrascal-Hernández D.C., Martínez-Cano J.P., Rodríguez Macías J.D., Grande-Tovar C.D. (2025). Evolution in Bone Tissue Regeneration: From Grafts to Innovative Biomaterials. Int. J. Mol. Sci..

[34] Haugen H.J., Lyngstadaas S.P., Rossi F., Perale G. (2019). Bone Grafts: Which Is the Ideal Biomaterial?. J. Clin. Periodontol..

[35] Dahlin C., Linde A., Gottlow J., Nyman S. (1988). Healing of Bone Defects by Guided Tissue Regeneration. Plast. Reconstr. Surg..

[36] Urban I.A., Monje A. (2019). Guided Bone Regeneration in Alveolar Bone Reconstruction. Oral Maxillofac. Surg. Clin. N. Am..

[37] Tanvir M.A.H., Khaleque M.A., Kim G.-H., Yoo W.-Y., Kim Y.-Y. (2024). The Role of Bioceramics for Bone Regeneration: History, Mechanisms, and Future Perspectives. Biomimetics.

[38] Gou M., Wang H., Xie H., Song H. (2024). Macrophages in Guided Bone Regeneration: Potential Roles and Future Directions. Front. Immunol..

[39] Costa N.M.F., Yassuda D.H., Sader M.S., Fernandes G.V.O., Soares G.D.A., Granjeiro J.M. (2016). Osteogenic Effect of Tricalcium Phosphate Substituted by Magnesium Associated with Genderm^®^ Membrane in Rat Calvarial Defect Model. Mater. Sci. Eng. C.

[40] Kamadjaja D.B., Harijadi A., Soesilawati P., Wahyuni E., Maulidah N., Fauzi A., Rah Ayu F., Simanjuntak R., Soesanto R., Asmara D. (2017). Demineralized Freeze-Dried Bovine Cortical Bone: Its Potential for Guided Bone Regeneration Membrane. Int. J. Dent..

[41] Yamanaka J.S., Oliveira A.C., Bastos A.R., Fernandes E.M., Reis R.L., Correlo V.M., Shimano A.C. (2023). Collagen Membrane from Bovine Pericardium for Treatment of Long Bone Defect. J. Biomed. Mater. Res. B Appl. Biomater..

[42] Garcia V.G., Dall Agnol G.d.S., Campista C.C.C., Bury L.L., Ervolino E., Longo M., Mulinari-Santos G., Levin L., Theodoro L.H. (2024). Evaluation of Two Anorganic Bovine Xenogenous Grafts in Bone Healing of Critical Defect in Rats Calvaria. Braz. Dent. J..

[43] Theodoro L.H., Campista C.C.C., Bury L.L., de Souza R.G.B., Muniz Y.S., Longo M., Mulinari-Santos G., Ervolino E., Levin L., Garcia V.G. (2024). Comparison of Different Bone Substitutes in the Repair of Rat Calvaria Critical Size Defects: Questioning the Need for Alveolar Ridge Presentation. Quintessence Int..

[44] Ali M., Mohd Noor S.N.F., Mohamad H., Ullah F., Javed F., Abdul Hamid Z.A. (2024). Advances in Guided Bone Regeneration Membranes: A Comprehensive Review of Materials and Techniques. Biomed. Phys. Eng. Express.

[45] Mizraji G., Davidzohn A., Gursoy M., Gursoy U.K., Shapira L., Wilensky A. (2023). Membrane Barriers for Guided Bone Regeneration: An Overview of Available Biomaterials. Periodontol. 2000.

[46] Baumer GenDerm. https://www.baumer.com.br/en/produtos/genderm.

[47] Bighetti A.C.C., Cestari T.M., Paini S., Pomini K.T., Buchaim D.V., Ortiz R.C., Júnior R.S.F., Barraviera B., Bullen I.R.F.R., Garlet G.P. (2024). Efficacy and Safety of a New Heterologous Fibrin Biopolymer on Socket Bone Healing after Tooth Extraction: An Experimental Pre-clinical Study. J. Clin. Periodontol..

[48] Ferreira R.S., Silva D.A.F.d., Biscola N.P., Sartori M.M.P., Denadai J.C., Jorge A.M., Santos L.D.d., Barraviera B. (2019). Traceability of Animal Protein Byproducts in Ruminants by Multivariate Analysis of Isotope Ratio Mass Spectrometry to Prevent Transmission of Prion Diseases. J. Venom. Anim. Toxins Incl. Trop. Dis..

[49] de Pontes L.G., Cavassan N.R.V., de Barros L.C., Ferreira Junior R.S., Barraviera B., Santos L.D. (2017). dos Plasma Proteome of Buffaloes. Proteom. Clin. Appl..

[50] Ferreira A.S.S.B.S., Barraviera B., Barraviera S.R.C.S., Abbade L.P.F., Caramori C.A., Ferreira R.S. (2013). A Success in Toxinology Translational Research in Brazil: Bridging the Gap. Toxicon.

[51] Barros L., Soares A., Costa F., Rodrigues V., Fuly A., Giglio J., Gallacci M., Thomazini-Santos I., Barraviera S., Barraviera B. (2011). Biochemical and Biological Evaluation of Gyroxin Isolated from *Crotalus Durissus Terrificus* Venom. J. Venom. Anim. Toxins Incl. Trop. Dis..

[52] Braud S. (2000). Snake Venom Proteins Acting on Hemostasis. Biochimie.

[53] Chiquito G.C.M. (2007). Comparison between Suture and Fibrin Adhesive Derived from Snake Venom for Fixation of Connective Tissue Graft in Correction of Marginal Tissue Recession. J. Venom. Anim. Toxins Incl. Trop. Dis..

[54] Spotnitz W.D. (2010). Fibrin Sealant: Past, Present, and Future: A Brief Review. World J. Surg..

[55] Thomazini-Santos I.A., Barraviera S.R.C.S., Mendes-Giannini M.J.S., Barraviera B. (2001). Surgical Adhesives. J. Venom. Anim. Toxins.

[56] Thomazini-Santos I.A., Giannini M.J.S.M., Toscano E., Machado P.E.A., Lima C.R.G., Barraviera B. (1998). The evaluation of clotting time in bovine thrombin, Reptilase ^®^, and thrombin-like fraction of *Crotalus durissus terrificus* venom using bovine, equine, ovine, bubaline and human cryoprecipitates. J. Venom. Anim. Toxins.

[57] Abbade L.P.F., Ferreira R.S., Santos L.D.d., Barraviera B. (2020). Chronic Venous Ulcers: A Review on Treatment with Fibrin Sealant and Prognostic Advances Using Proteomic Strategies. J. Venom. Anim. Toxins Incl. Trop. Dis..

[58] de Oliveira Gonçalves J.B., Buchaim D.V., de Souza Bueno C.R., Pomini K.T., Barraviera B., Júnior R.S.F., Andreo J.C., de Castro Rodrigues A., Cestari T.M., Buchaim R.L. (2016). Effects of Low-Level Laser Therapy on Autogenous Bone Graft Stabilized with a New Heterologous Fibrin Sealant. J. Photochem. Photobiol. B.

[59] Ferreira R.S., de Barros L.C., Abbade L.P.F., Barraviera S.R.C.S., Silvares M.R.C., de Pontes L.G., dos Santos L.D., Barraviera B. (2017). Heterologous Fibrin Sealant Derived from Snake Venom: From Bench to Bedside—An Overview. J. Venom. Anim. Toxins Incl. Trop. Dis..

[60] Troeltzsch M., Troeltzsch M., Kauffmann P., Gruber R., Brockmeyer P., Moser N., Rau A., Schliephake H. (2016). Clinical Efficacy of Grafting Materials in Alveolar Ridge Augmentation: A Systematic Review. J. Cranio-Maxillofac. Surg..

[61] Buchaim D.V., Rodrigues A.d.C., Buchaim R.L., Barraviera B., Junior R.S.F., Junior G.M.R., Bueno C.R.d.S., Roque D.D., Dias D.V., Dare L.R. (2016). The New Heterologous Fibrin Sealant in Combination with Low-Level Laser Therapy (LLLT) in the Repair of the Buccal Branch of the Facial Nerve. Lasers Med. Sci..

[62] Farivar S., Malekshahabi T., Shiari R. (2014). Biological Effects of Low Level Laser Therapy. J. Lasers Med. Sci..

[63] Ferreira F.N.H., Gondim J.O., Neto J.J.S.M., dos Santos P.C.F., de Freitas Pontes K.M., Kurita L.M., de Araújo M.W.A. (2016). Effects of Low-Level Laser Therapy on Bone Regeneration of the Midpalatal Suture after Rapid Maxillary Expansion. Lasers Med. Sci..

[64] Rosso M.P.d.O., Buchaim D.V., Pomini K.T., Coletta B.B.D., Reis C.H.B., Pilon J.P.G., Duarte Júnior G., Buchaim R.L. (2019). Photobiomodulation Therapy (PBMT) Applied in Bone Reconstructive Surgery Using Bovine Bone Grafts: A Systematic Review. Materials.

[65] Theodoro L.H., Caiado R.C., Longo M., Novaes V.C.N., Zanini N.A., Ervolino E., de Almeida J.M., Garcia V.G. (2015). Effectiveness of the Diode Laser in the Treatment of Ligature-Induced Periodontitis in Rats: A Histopathological, Histometric, and Immunohistochemical Study. Lasers Med. Sci..

[66] Ahrari F., Shafaee H., Haghpanahi M., Bardideh E. (2024). Low-Level Laser Therapy and Laser Acupuncture Therapy for Pain Relief after Initial Archwire Placement. J. Orofac. Orthop./Fortsch. Kieferorthopädie.

[67] de Freitas L.C., Kim D.S., Santana da Costa D., Fernandes Soutello H.P., Salata T.R., Sato L.F., Takahashi N.I., de Souza Gomes V., Kondo P.T., Lomonaco G.G. (2025). The Role of Photobiomodulation in the Functional Recovery of Proximal Humerus Fractures: A Randomized Controlled Clinical Protocol. PLoS ONE.

[68] Yong J., Gröger S., Von Bremen J., Martins Marques M., Braun A., Chen X., Ruf S., Chen Q. (2023). Photobiomodulation Therapy Assisted Orthodontic Tooth Movement: Potential Implications, Challenges, and New Perspectives. J. Zhejiang Univ.-Sci. B.

[69] Kulkarni S., George R., Love R., Ranjitkar S. (2022). Effectiveness of Photobiomodulation in Reducing Pain and Producing Dental Analgesia: A Systematic Review. Lasers Med. Sci..

[70] Petridis X., Diamanti E., Trigas G.C., Kalyvas D., Kitraki E. (2015). Bone Regeneration in Critical-Size Calvarial Defects Using Human Dental Pulp Cells in an Extracellular Matrix-Based Scaffold. J. Cranio-Maxillofac. Surg..

[71] Abou Fadel R., Samarani R., Chakar C. (2018). Guided Bone Regeneration in Calvarial Critical Size Bony Defect Using a Double-Layer Resorbable Collagen Membrane Covering a Xenograft: A Histological and Histomorphometric Study in Rats. Oral Maxillofac. Surg..

[72] Saki M., Tahamtan S., Shavakhi M., Grzech-Leśniak K., Fekrazad R. (2025). The Effectiveness of Photobiomodulation Therapy on Bone Regeneration of Oral and Craniofacial Defects. A Systematic Review of Animal, and in-Vitro Studies. Lasers Med. Sci..

[73] Li S., Dan X., Chen H., Li T., Liu B., Ju Y., Li Y., Lei L., Fan X. (2024). Developing Fibrin-Based Biomaterials/Scaffolds in Tissue Engineering. Bioact. Mater..

[74] Noori A., Ashrafi S.J., Vaez-Ghaemi R., Hatamian-Zaremi A., Webster T.J. (2017). A Review of Fibrin and Fibrin Composites for Bone Tissue Engineering. Int. J. Nanomed..

[75] Ramires G.A.D., Helena J.T., Oliveira J.C.S.D., Faverani L.P., Bassi A.P.F. (2021). Evaluation of Guided Bone Regeneration in Critical Defects Using Bovine and Porcine Collagen Membranes: Histomorphometric and Immunohistochemical Analyses. Int. J. Biomater..

[76] Titsinides S., Agrogiannis G., Karatzas T. (2019). Bone Grafting Materials in Dentoalveolar Reconstruction: A Comprehensive Review. Jpn. Dent. Sci. Rev..

[77] Retzepi M., Donos N. (2010). Guided Bone Regeneration: Biological Principle and Therapeutic Applications. Clin. Oral Implants Res..

[78] Mistry A.S., Mikos A.G. (2005). Tissue Engineering Strategies for Bone Regeneration. Adv. Biochem. Eng. Biotechnol..

[79] Miron R.J., Zhang Y.F. (2012). Osteoinduction. J. Dent. Res..

[80] Valiati R., Paes J.V., de Moraes A.N., Gava A., Agostini M., Masiero A.V., de Oliveira M.G., Pagnoncelli R.M. (2012). Effect of Low-Level Laser Therapy on Incorporation of Block Allografts. Int. J. Med. Sci..

[81] Garcia V.J., Arnabat J., Comesaña R., Kasem K., Ustrell J.M., Pasetto S., Segura O.P., ManzanaresCéspedes M.C., Carvalho-Lobato P. (2016). Effect of Low-Level Laser Therapy after Rapid Maxillary Expansion: A Clinical Investigation. Lasers Med. Sci..

[82] de Oliveira G.J.P.L., Aroni M.A.T., Medeiros M.C., Marcantonio E., Marcantonio R.A.C. (2018). Effect of Low-level Laser Therapy on the Healing of Sites Grafted with Coagulum, Deproteinized Bovine Bone, and Biphasic Ceramic Made of Hydroxyapatite and Β-tricalcium Phosphate. In Vivo Study in Rats. Lasers Surg. Med..

[83] Varela P.J.R., Barros P.A.G., Montagner P.G., Provout M.B., Martinez E.F., Suzuki S.S., Garcez A.S. (2023). Can Collagen Membrane on Bone Graft Interfere with Light Transmission and Influence Tissue Neoformation During Photobiomodulation? A Preliminary Study. Photobiomodul Photomed. Laser Surg..

[84] Freitas N.R.d., Guerrini L.B., Esper L.A., Sbrana M.C., Dalben G.d.S., Soares S., Almeida A.L.P.F.d. (2018). Evaluation of Photobiomodulation Therapy Associated with Guided Bone Regeneration in Critical Size Defects. In Vivo Study. J. Appl. Oral. Sci..

[85] Della Coletta B.B., Jacob T.B., Moreira L.A.d.C., Pomini K.T., Buchaim D.V., Eleutério R.G., Pereira E.d.S.B.M., Roque D.D., Rosso M.P.d.O., Shindo J.V.T.C. (2021). Photobiomodulation Therapy on the Guided Bone Regeneration Process in Defects Filled by Biphasic Calcium Phosphate Associated with Fibrin Biopolymer. Molecules.

[86] Rufato F.C.T., de Sousa L.G., Scalize P.H., Gimenes R., Regalo I.H., Rosa A.L., Beloti M.M., de Oliveira F.S., Bombonato-Prado K.F., Regalo S.C.H. (2022). Texturized P(VDF-TrFE)/BT Membrane Enhances Bone Neoformation in Calvaria Defects Regardless of the Association with Photobiomodulation Therapy in Ovariectomized Rats. Clin. Oral Investig..

[87] Emrem Doğan G., Demir T., Orbak R. (2014). Effect of Low-Level Laser on Guided Tissue Regeneration Performed with Equine Bone and Membrane in the Treatment of İntrabony Defects: A Clinical Study. Photomed. Laser Surg..

[88] Luca R.E., Giuliani A., Mănescu A., Heredea R., Hoinoiu B., Constantin G.D., Duma V.-F., Todea C.D. (2020). Osteogenic Potential of Bovine Bone Graft in Combination with Laser Photobiomodulation: An Ex Vivo Demonstrative Study in Wistar Rats by Cross-Linked Studies Based on Synchrotron Microtomography and Histology. Int. J. Mol. Sci..

[89] Freitas N.R.d., Guerrini L.B., Esper L.A., Sbrana M.C., Santos C.C.V.d., Almeida A.L.P.F.d. (2023). Photobiomodulation and Inorganic Bovine Bone in Guided Bone Regeneration: Histomorphometric Analysis in Rats. J. Funct. Biomater..

[90] Pinheiro A.L.B., Martinez Gerbi M.E., de Assis Limeira F., Carneiro Ponzi E.A., Marques A.M.C., Carvalho C.M., de Carneiro Santos R., Oliveira P.C., Nóia M., Ramalho L.M.P. (2009). Bone Repair Following Bone Grafting Hydroxyapatite Guided Bone Regeneration and Infra-Red Laser Photobiomodulation: A Histological Study in a Rodent Model. Lasers Med. Sci..

[91] Chiari S. (2016). Photobiomodulation and Lasers. Front. Oral. Biol..

[92] Mosca R.C., Ong A.A., Albasha O., Bass K., Arany P. (2019). Photobiomodulation Therapy for Wound Care: A Potent, Noninvasive, Photoceutical Approach. Adv. Skin. Wound Care.

[93] Lopes C.d.C.A., Limirio J.P.J.O., Zanatta L.S.A., Simamoto V.R.N., Dechichi P., Limirio P.H.J.O. (2022). Effectiveness of Photobiomodulation Therapy on Human Bone Healing in Dentistry: A Systematic Review. Photobiomodul Photomed. Laser Surg..

[94] Berni M., Brancato A.M., Torriani C., Bina V., Annunziata S., Cornella E., Trucchi M., Jannelli E., Mosconi M., Gastaldi G. (2023). The Role of Low-Level Laser Therapy in Bone Healing: Systematic Review. Int. J. Mol. Sci..

[95] Sella V.R.G., do Bomfim F.R.C., Machado P.C.D., da Silva Morsoleto M.J.M., Chohfi M., Plapler H. (2015). Effect of Low-Level Laser Therapy on Bone Repair: A Randomized Controlled Experimental Study. Lasers Med. Sci..

[96] de Lima Luna C.A., do Couto M.F.N., Alves M.S.A., de Andrade Hage C., de Figueiredo Chaves R.H., Guimarães D.M. (2025). Photobiomodulation of Alveolar Bone Healing in Rats with Low-Level Laser and Light Emitting Diode Therapy. Lasers Med. Sci..

[97] Hazrati P., Azadi A., Fekrazad S., Wang H.-L., Fekrazad R. (2025). The Effect of Photobiomodulation Therapy on Fracture Healing: A Systematic Review and Meta-Analysis of Animal Studies. Lasers Med. Sci..

[98] Bossini P.S., Rennó A.C.M., Ribeiro D.A., Fangel R., Ribeiro A.C., Lahoz M.d.A., Parizotto N.A. (2012). Low Level Laser Therapy (830 nm) Improves Bone Repair in Osteoporotic Rats: Similar Outcomes at Two Different Dosages. Exp. Gerontol..

[99] Tim C.R., Bossini P.S., Kido H.W., Malavazi I., von Zeska Kress M.R., Carazzolle M.F., Rennó A.C., Parizotto N.A. (2016). Low-Level Laser Therapy Induces an Upregulation of Collagen Gene Expression during the Initial Process of Bone Healing: A Microarray Analysis. J. Biomed. Opt..

[100] Tim C.R., Bossini P.S., Kido H.W., Malavazi I., von Zeska Kress M.R., Carazzolle M.F., Parizotto N.A., Rennó A.C. (2016). Effects of Low Level Laser Therapy on Inflammatory and Angiogenic Gene Expression during the Process of Bone Healing: A Microarray Analysis. J. Photochem. Photobiol. B.

[101] Cunha J.L.S., de Carvalho F.M.d.A., Pereira Filho R.N., Ribeiro M.A.G., de Albuquerque-Júnior R.L.C. (2019). Effects of Different Protocols of Low-Level Laser Therapy on Collagen Deposition in Wound Healing. Braz. Dent. J..

[102] Dimitriou R., Mataliotakis G.I., Calori G.M., Giannoudis P. (2012). V The Role of Barrier Membranes for Guided Bone Regeneration and Restoration of Large Bone Defects: Current Experimental and Clinical Evidence. BMC Med..

[103] Elgali I., Omar O., Dahlin C., Thomsen P. (2017). Guided Bone Regeneration: Materials and Biological Mechanisms Revisited. Eur. J. Oral Sci..

[104] Ma C., Ye Y., Shi X., Li N., Chen Y., Shi X., Chen H. (2025). Photobiomodulation Promotes Osteogenic Differentiation of Mesenchymal Stem Cells and Increases P-Akt Levels in Vitro. Sci. Rep..

[105] Magri A.M.P., Parisi J.R., de Andrade A.L.M., Rennó A.C.M. (2021). Bone Substitutes and Photobiomodulation in Bone Regeneration: A Systematic Review in Animal Experimental Studies. J. Biomed. Mater. Res. A.

[106] Vigliar M.F.R., Marega L.F., Duarte M.A.H., Alcalde M.P., Rosso M.P.d.O., Ferreira Junior R.S., Barraviera B., Reis C.H.B., Buchaim D.V., Buchaim R.L. (2024). Photobiomodulation Therapy Improves Repair of Bone Defects Filled by Inorganic Bone Matrix and Fibrin Heterologous Biopolymer. Bioengineering.

[107] Veríssimo D.M., Leitão R.F., Figueiró S.D., Góes J.C., Lima V., Silveira C.O., Brito G.A. (2015). Guided Bone Regeneration Produced by New Mineralized and Reticulated Collagen Membranes in Critical-Sized Rat Calvarial Defects. Exp. Biol. Med..

[108] Amaroli A., Agas D., Laus F., Cuteri V., Hanna R., Sabbieti M.G., Benedicenti S. (2018). The Effects of Photobiomodulation of 808 Nm Diode Laser Therapy at Higher Fluence on the in Vitro Osteogenic Differentiation of Bone Marrow Stromal Cells. Front. Physiol..

[109] Tani A., Chellini F., Giannelli M., Nosi D., Zecchi-Orlandini S., Sassoli C. (2018). Red (635 Nm), Near-Infrared (808 Nm) and Violet-Blue (405 Nm) Photobiomodulation Potentiality on Human Osteoblasts and Mesenchymal Stromal Cells: A Morphological and Molecular In Vitro Study. Int. J. Mol. Sci..

[110] Torquato L.C., Suárez E.A.C., Bernardo D.V., Pinto I.L.R., Mantovani L.O., Silva T.I.L., Jardini M.A.N., Santamaria M.P., De Marco A.C. (2021). Bone Repair Assessment of Critical Size Defects in Rats Treated with Mineralized Bovine Bone (Bio-Oss^®^) and Photobiomodulation Therapy: A Histomorphometric and Immunohistochemical Study. Lasers Med. Sci..

[111] Hjazi A. (2025). A Collagen-Based Amniotic Membrane Scaffold Combined with Photobiomodulation Accelerates Wound Repair in Diabetic Rats through Modulation of Inflammation and Tissue Regeneration. Tissue Cell.

[112] Karimi M.R., Abdollahi S., Etemadi A., Hakimiha N. (2024). Investigating the Effect of Photobiomodulation Therapy With Different Wavelengths of Diode Lasers on the Proliferation and Adhesion of Human Gingival Fibroblast Cells to a Collagen Membrane: An In Vitro Study. J. Lasers Med. Sci..

[113] Surmeli Baran S., Temmerman A., Salimov F., Ucak Turer O., Sapmaz T., Haytac M.C., Ozcan M. (2021). The Effects of Photobiomodulation on Leukocyte and Platelet-Rich Fibrin as Barrier Membrane on Bone Regeneration: An Experimental Animal Study. Photobiomodul Photomed. Laser Surg..

[114] Alves F.A.M., Marques M.M., Cavalcanti S.C.S.X.B., Pedroni A.C.F., Ferraz E.P., Miniello T.G., Moreira M.S., Jerônimo T., Deboni M.C.Z., Lascala C.A. (2020). Photobiomodulation as Adjunctive Therapy for Guided Bone Regeneration. A MicroCT Study in Osteoporotic Rat Model. J. Photochem. Photobiol. B.

[115] Moscatel M.B.M., Pagani B.T., Trazzi B.F.d.M., Reis C.H.B., Ribeiro C.A., Buchaim D.V., Buchaim R.L. (2025). Effects of Photobiomodulation in Association with Biomaterials on the Process of Guided Bone Regeneration: An Integrative Review. Ceramics.

[116] Shaikh-Kader A., Houreld N.N. (2022). Photobiomodulation, Cells of Connective Tissue and Repair Processes: A Look at In Vivo and In Vitro Studies on Bone, Cartilage and Tendon Cells. Photonics.

[117] Oury F. (2012). A Crosstalk between Bone and Gonads. Ann. N. Y Acad. Sci..

[118] Venken K., Callewaert F., Boonen S., Vanderschueren D. (2008). Sex Hormones, Their Receptors and Bone Health. Osteoporos. Int..

[119] de Assis Limeira F., Barbosa Pinheiro A., Marquez de Martinez Gerbi M., Pedreira Ramalho L., Marzola C., Carneiro Ponzi E., Soares A., Bandeira de Carvalho L., Vieira Lima H., Oliveira Gonçalves T. (2003). Assessment of bone repair following the use of anorganic bone graft and membrane associated or not to 830-nm laser light. Lasers Dent. IX.

[120] Buchaim D.V., Cassaro C.V., Shindo J.V.T.C., Coletta B.B.D., Pomini K.T., Rosso M.P.d.O., Campos L.M.G., Ferreira R.S., Barraviera B., Buchaim R.L. (2019). Unique Heterologous Fibrin Biopolymer with Hemostatic, Adhesive, Sealant, Scaffold and Drug Delivery Properties: A Systematic Review. J. Venom. Anim. Toxins Incl. Trop. Dis..

[121] Le Guéhennec L., Layrolle P., Daculsi G. (2004). A Review of Bioceramics and Fibrin Sealant. Eur. Cell Mater..

[122] Barbosa M.D.S., Gregh S.L.A., Passanezi E. (2007). Fibrin Adhesive Derived From Snake Venom in Periodontal Surgery. J. Periodontol..

[123] Biscola N.P., Cartarozzi L.P., Ulian-Benitez S., Barbizan R., Castro M.V., Spejo A.B., Ferreira R.S., Barraviera B., Oliveira A.L.R. (2017). Multiple Uses of Fibrin Sealant for Nervous System Treatment Following Injury and Disease. J. Venom. Anim. Toxins Incl. Trop. Dis..

[124] Cunha M.R.d., Menezes F.A., Santos G.R.d., Pinto C.A.L., Barraviera B., Martins V.d.C.A., Plepis A.M.d.G., Ferreira Junior R.S. (2015). Hydroxyapatite and a New Fibrin Sealant Derived from Snake Venom as Scaffold to Treatment of Cranial Defects in Rats. Mater. Res..

[125] Percie du Sert N., Ahluwalia A., Alam S., Avey M.T., Baker M., Browne W.J., Clark A., Cuthill I.C., Dirnagl U., Emerson M. (2020). Reporting Animal Research: Explanation and Elaboration for the ARRIVE Guidelines 2.0. PLoS Biol..

[126] Tominaga H., Kawamura I., Tokumoto H., Tawaratsumida H., Ogura T., Kuroshima T., Ijiri K., Taniguchi N. (2024). Fibrin Glue-Coated Collagen Matrix Is Superior to Fibrin Glue-Coated Polyglycolic Acid for Preventing Cerebral Spinal Fluid Leakage after Spinal Durotomy. Sci. Rep..

[127] Ciardulli M.C., Lovecchio J., Parolini O., Giordano E., Maffulli N., Della Porta G. (2024). Fibrin Scaffolds Perfused with Transforming Growth Factor-Β1 as an In Vitro Model to Study Healthy and Tendinopathic Human Tendon Stem/Progenitor Cells. Int. J. Mol. Sci..

[128] Kenny S., Gabra H., Hall N.J., Flageole H., Illie B., Barnett E., Kocharian R., Sharif K. (2024). A Study of Safety and Effectiveness of Evicel Fibrin Sealant as an Adjunctive Hemostat in Pediatric Surgery. Eur. J. Pediatr. Surg..

[129] Schröger S.-V., Blatt S., Sagheb K., Al-Nawas B., Kämmerer P.W., Sagheb K. (2024). Platelet-Rich Fibrin for Rehydration and Pre-Vascularization of an Acellular, Collagen Membrane of Porcine Origin. Clin. Oral Investig..

